# Bullous Pemphigoid Causing Successive Emergency Department Visits

**DOI:** 10.5811/cpcem.1415

**Published:** 2023-10-27

**Authors:** Edmund Hsu, Andrew T. Kinoshita, C. Eric McCoy

**Affiliations:** * University of California Irvine, Department of Emergency Medicine, Orange, California; † University of California Irvine, School of Medicine, Orange, California

**Keywords:** bullous pemphigoid, bullae, blister, pruritus

## Abstract

**Case Presentation:**

In this case presentation, an 84-year-old male with Fitzpatrick type IV skin tone experienced blistering due to bullous pemphigoid (BP), first on the distal upper left extremity and then on the distal lower extremities, chest, and back. These symptoms resulted in three visits to the emergency department within a month, as well as an episode of hospitalization. Despite treatment, the blistering did not resolve until future outpatient care with dermatology.

**Discussion:**

Bullous pemphigoid is a rare autoimmune disease where autoantibodies target hemidesmosomal proteins causing basement membrane destruction and tense subepithelial bullae with pruritus. While uncommon, the incidence of BP is increasing. Bullous pemphigoid tends to affect older adults, appearing as a rash prior to bullae formation on the abdomen, extremities, groin, axillae, or mucosa. Bullous pemphigoid may also be drug-related with atypical symptoms. Diagnosis of BP should be based on immunopathology, and initial treatment of BP is through corticosteroid or doxycycline.

CPC-EM Capsule What do we already know about this clinical entity?

*In bullous pemphigoid (BP) autoantibodies target hemidesmosomal proteins causing basement membrane destruction and subepithelial bullae with pruritus.*

What makes this presentation of disease reportable?

*An 84-year-old male with Fitzpatrick type IV skin tone experienced BP that spread across multiple regions and resulted in three ED visits.*

What is the major learning point?

*This case, showing images of BP on darker skin tone, demonstrates how the severity of the condition may evolve despite treatment.*

How might this improve emergency medicine practice?

*The case aids in recognition of BP and offers a quick guide to initial treatment as well as insight into the possible persistence of the condition.*

## CASE PRESENTATION

**Image 1.**
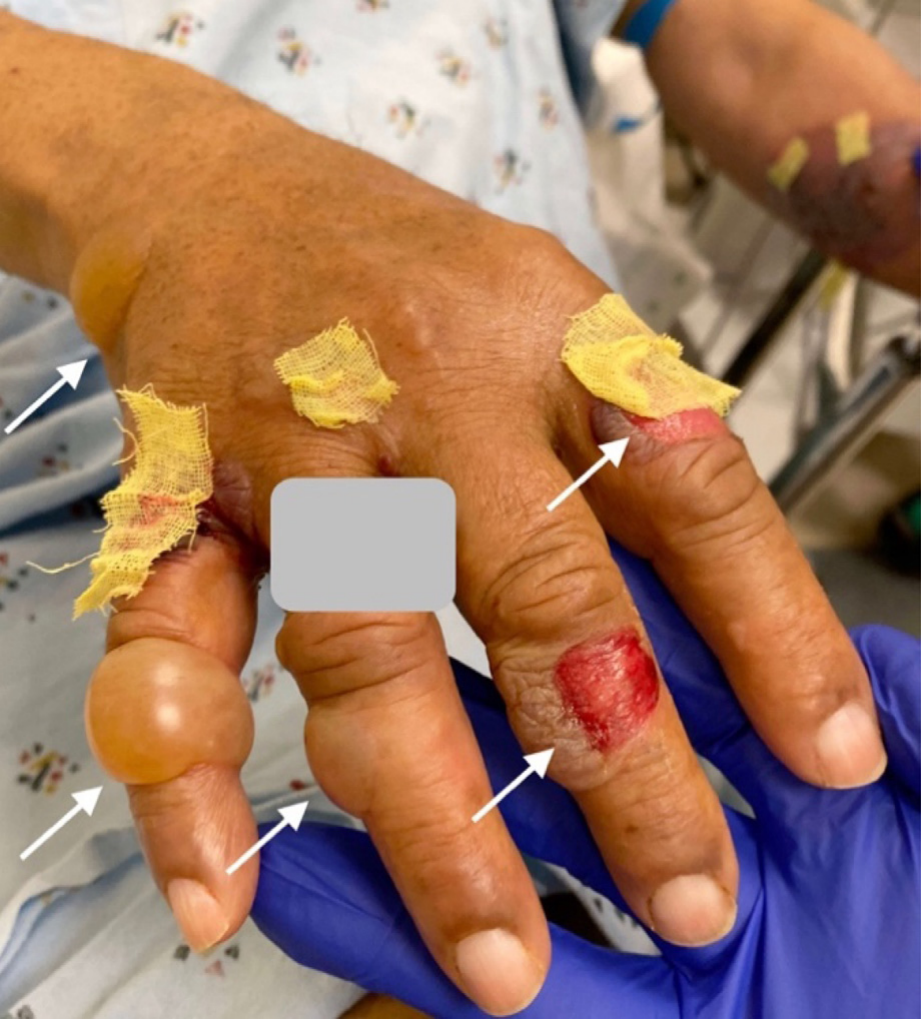
Dorsal aspect of hand with bullous pemphigoid blisters, some ruptured; arrows indicate blisters. (Gray box covers patient identifier.)

**Image 2.**
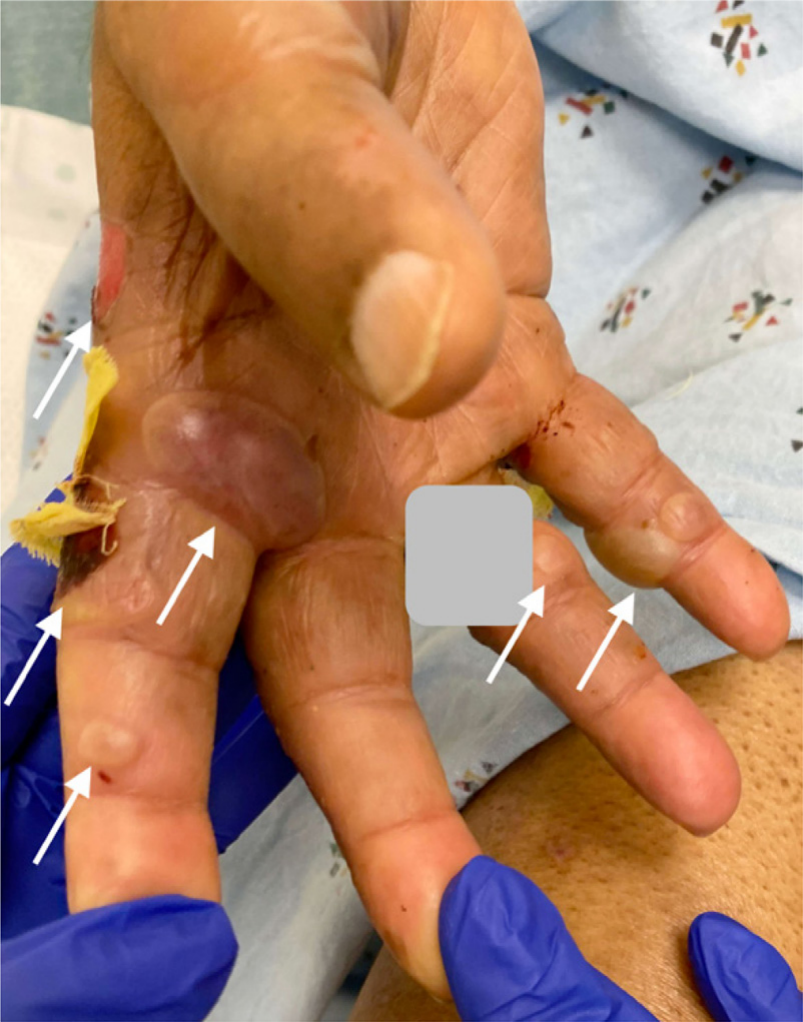
Ventral aspect of hand with bullous pemphigoid, some ruptured; arrows indicate blisters. (Gray box covers patient identifier.)

**Image 3.**
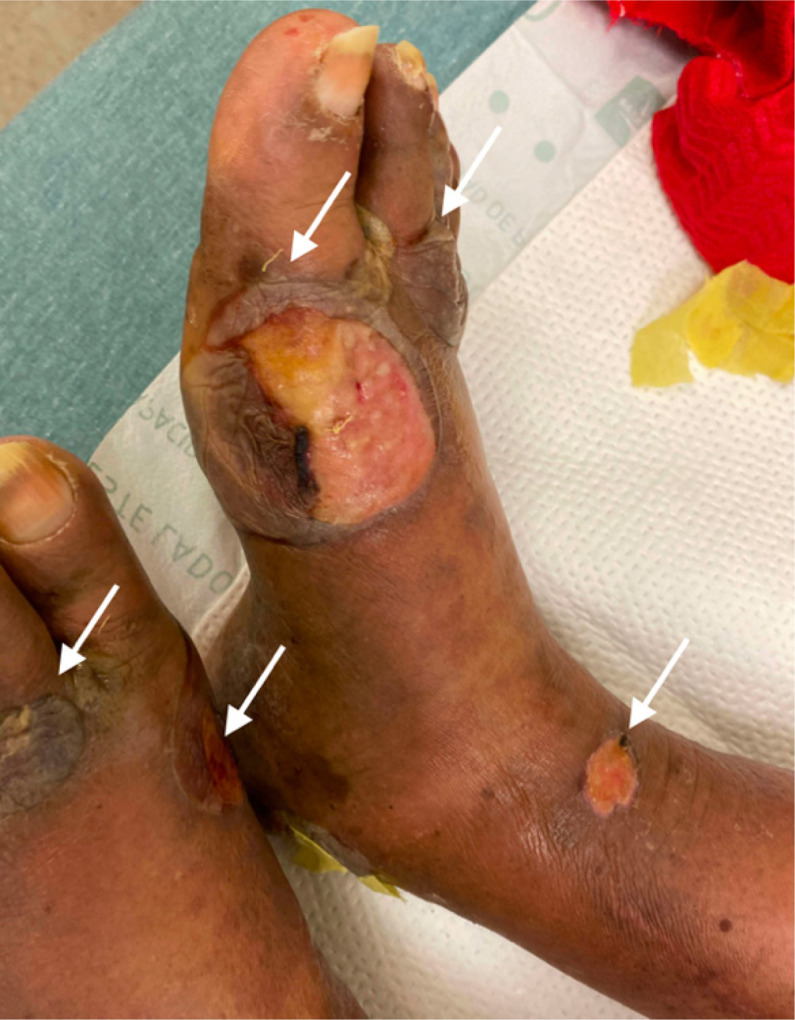
Foot and ankle with bullous pemphigoid, some ruptured. Arrows indicate blisters.

An 84-year-old male with a history of diabetes mellitus, Alzheimer’s disease, Parkinson’s disease, and coronary artery disease with previous coronary artery bypass graft presented to the emergency department (ED) with hyperglycemia and blistering on his distal upper and lower extremities at multiple stages of healing (Images 1–3). Four weeks prior, the patient went to the ED for painless pruritic blistering on the left upper extremity that had spread toward the axilla and groin over a period of two months. He was treated for presumptive bullous pemphigoid (BP) and discharged on doxycycline and clobetasol. Symptoms improved until two weeks following ED discharge, and the patient returned to the ED for now painful blistering of his distal lower extremities, as well as blistering of the chest and back. He was admitted to the hospital for three days and seen by dermatology, who entertained the diagnosis of bullous lymphedema versus bullous diabeticorum. A biopsy was not performed due to concern for poor wound healing. Leg compression and elevation was recommended upon discharge.

The patient returned to the ED one week after discharge due to ongoing blisters and extremity swelling. At this visit, vital signs were stable with most recent hemoglobin A1C elevated at 10.0% (normal below 5.7%; prediabetes 5.7%–6.4%; diabetes above 6.5%). Dermatology was consulted and recommended outpatient evaluation. Insulin was modified, and the patient was discharged. Diagnosis of BP was confirmed at later outpatient evaluation through positive tests for BP antibodies. The patient responded to prednisone on an oral treatment regimen of 60 milligrams (mg) daily for 30 days, tapered to 40 mg for three weeks and then 20 mg for three weeks.

## DISCUSSION

Bullous pemphigoid is a rare autoimmune disease in which autoantibodies target hemidesmosomal proteins dystonin-e (BP antigen 1 or BP230) and collagen XVII (BP antigen 2 or BP180), causing basement membrane destruction and tense subepithelial bullae with pruritus.^1^ ^,^ ^2^ Incidence is increasing with estimates in the United States and European states between 10-43 per one million individuals per year.^2^ ^,^ ^3^ Classically, BP affects older adults, sometimes appearing as a rash before bullae of 1–3 centimeters (cm) diameter appear.^1^ The images presented here demonstrate BP of moderate severity on light brown skin tone (Fitzpatrick type IV). Distribution is often symmetric and commonly affects the abdomen, extremities, groin, axillae, or mucosa.^1^ The disease is usually chronic with exacerbations and remissions.^1^

Bullous pemphigoid is associated with some neurologic disorders, including dementia and Parkinson’s disease.^2^ Atypical presentations of BP include non-bullous pemphigoid, which accounts for 20% of cases.^2^ Additionally, BP has been associated with many classes of drugs, and this may also cause atypical presentations, including younger age of onset or blisters without initial rash.^4^ Drug-related BP may resolve fully with cessation of the offending agent.^1^ Diagnostic studies used in the evaluation of patients with lesions suspicious of BP include biopsy (for histopathologic examination and direct immunofluorescence microscopy) and serum tests to detect circulating anti-basement membrane zone antibodies.^1^ ^,^ ^2^ The mainstays of initial treatment for this condition include corticosteroids and doxycycline.^1^ ^,^ ^5^
